# Metabolic syndrome increases operative mortality in patients with impaired left ventricular systolic function who undergo coronary artery bypass grafting: a retrospective observational study

**DOI:** 10.1186/s12872-019-1004-8

**Published:** 2019-01-23

**Authors:** Shuangkun Chen, Jiahui Li, Qianzhen Li, Zhihuang Qiu, Xijie Wu, Liangwan Chen

**Affiliations:** Department of Cardiac Surgery, Union Hospital, Fujian Medical University, Fuzhou, 350001 Fujian China

**Keywords:** Metabolic syndrome, Left ventricular dysfunction, Mortality, CABG

## Abstract

**Background:**

Metabolic syndrome (MetS) is a prevalent risk factor for coronary artery disease progression. Past studies have shown that MetS and its components tends to increase mortality after coronary artery bypass grafting (CABG), but data on the impact of MetS on postoperative outcome in patients with a left ventricular (LV) ejection fraction (EF) < 50% are still lacking.

**Methods:**

Out of 2300 patients who underwent CABG between 2008 and 2018 in our center, 190 patients were identified as having impaired LV systolic function (EF < 50%). The patients were divided into two groups: those with MetS (*n* = 87, 45.8%) and those without MetS (*n* = 103, 54.2%). The influence of MetS on postoperative mortality and major complications was investigated.

**Results:**

Postoperative mortality occurred in 12.6% of patients with MetS and in 3.9% of patients without MetS (*p* < 0.05). Multivariate analysis showed that patients with MetS had a significantly greater risk of mortality compared with patients without MetS (relative risk 7.23, *p* < 0.05). After adjustment for other risk factors, the risk of mortality was increased 6.47-fold [95% confidence interval (CI):1.25–33.6; *p* < 0.05] in patients with MetS and diabetes and 5.4-fold (95% CI: 1.12–29.7; *p* < 0.05) in patients with MetS and without diabetes, whereas it was not significantly increased in patients with diabetes and without MetS.

**Conclusions:**

MetS is an important predictor of increased mortality in patients with LVEF<50% who undergo CABG. The components of MetS have synergistic effect in postoperative mortality. Multifactorial intervention in MetS is required to improve surgical efficacy in these patients.

## Introduction

Diabetes mellitus (DM) and obesity are increasingly prevalent in the population undergoing coronary artery bypass grafting (CABG), and these conditions are major factors in the development of metabolic syndrome (MetS) [1–4]. MetS is a clustering of risk factors, including obesity (especially visceral adiposity), diabetes or impaired glucose regulation, lipid disorders and increased blood pressure, that leads to a dramatic increase in the risk of cardiovascular disease (CVD) [5, 6]. CVD is the leading cause of death among the population with these risk factors [7]. Several studies have reported poor prognosis after myocardial infarction (MI) that might be triggered by the unfavorable effects of MetS on left ventricular (LV) function [8–10]. MetS may lead to greater infarct size, resulting in LV dysfunction [11]. Past clinical studies have demonstrated that MetS and its components are independent risk factor for operative morbidity and mortality after CABG surgery [6, 7, 12–14]. However, the prevalence of MetS among patients with LV ejection fractions (EF) < 50% who undergo CABG and the impact of MetS on postoperative clinical outcomes are still unknown. The aim of this retrospective study was to confirm the effects of MetS on patients with impaired LV systolic function after CABG surgery.

## Materials and methods

### Study population

Subjects were selected from a total of 2300 patients who underwent CABG at our institution between January 1, 2008, and May 31, 2018. Echocardiographic results were obtained within 2 weeks prior to surgery in all patients. Among these patients, we identified 200 patients who had an LVEF of 50% or less in preoperative echocardiographic evaluations. In all, 10 patients were excluded from the study because of missing data in our database. Of the included patients, 87 (45.8%) met the criteria for MetS and were referred to as the MetS group, and 103 patients (54.2%) who did not were referred to as the non-MetS group. This study was approved by our institutional Ethics Committee, which waived the requirement for informed patient consent because of the retrospective nature of the study.

### Surgical technique

During the study period, a team of 9 surgeons performed the CABG procedures. The surgeons adopted different surgical strategies for CABG (off-pump vs. on-pump). On-pump CABG was preferred in patients with unstable hemodynamics or complicating valvular disease or other cardiac malformations. Median sternotomy was performed in all cases. The internal mammary artery (IMA) and/or saphenous vein were harvested using standard techniques.

For off-pump CABG, 3 traction sutures were placed in the posterior pericardium for retraction. Regional myocardial immobilization was achieved with a suction stabilizer (Guidant Axius Coronary Shunt, Guidant, CA, USA) during distal anastomosis, and intracoronary shunts (Chase Medical, Richardson, TX, USA) of appropriate sizes were used to maintain flow to the distal myocardium during the anastomosis. A nontraumatic small bulldog clamp was then applied to the target vessel proximal to the anastomotic site to achieve hemostasis after arteriotomy. For on-pump CABG, all procedures were performed through a standard cardiopulmonary bypass. When patients were cooled to 30 °C (moderate hypothermia), the ascending aorta was cross-clamped, and myocardial preservation was achieved with anterograde tepid blood cardioplegia. All anastomosis were constructed with a continuous-suture technique with 7–0 or 8–0 monofilament sutures.

After the operation, patients were taken to a dedicated cardiovascular intensive care unit (ICU). They were extubated if they met the following criteria: clear consciousness, hemodynamic stability, recovery of myodynamia and no significant bleeding.

### Identification

Patients were considered to have MetS when 3 or more of the following 5 criteria were present, based on a modification of the National Cholesterol Education Program Expert Panel on Detection, Evaluation, and Treatment of High Blood Cholesterol in Adults (Adult Treatment Panel III) (NCEP-ATP III): 1) obesity, defined as a body mass index (BMI) > 25 kg/m^2^ based on the established Chinese criteria for obesity; 2) fasting glycemia ≥6.1 mmol/l or treatment with oral hypoglycemics or insulin; 3) triglycerides ≥1.69 mmol/l; 4) high-density lipoprotein (HDL) cholesterol < 1.04 mmol/l in men and < 1.29 mmol/l in women; and 5) blood pressure > 130/85 mmHg or treatment with antihypertensive medication. The original criterion to classify obesity in the NCEP-ATP III was a waist circumference > 120 cm in men or > 88 cm in women, but this was not measured in this cohort; therefore, we used BMI. Recent studies have shown that most patients identified as having MetS based on BMI would also have been diagnosed as obese according to waist circumference [15, 16].

Perioperative myocardial infarction (MI) was defined as an increase in creatinine kinase-MB (CK-MB) to ≥10 times the upper limit of normal or ≥ 5times the upper limit of normal with new 30-ms Q waves within 24 h of surgery. The criterion for postoperative renal failure was defined as a need for dialysis to treat prolonged oliguria or anuria. The presence of a positive blood culture and signs and symptoms consistent with sepsis was used to define septicemia. Stroke was defined as central neurological deficit persisting for more than 72 h. Atrial fibrillation was defined as the occurrence of any atrial fibrillation during hospitalization. Low cardiac output syndrome (LCOS) was defined as the use of inotropic agents or intra-aortic balloon pump support, or both, to maintain a cardiac index > 2.0 L·min^− 1^·m^2^.

### Statistical analysis

Continuous variables are described as the means ± standard deviation, and categorical data are expressed as frequencies and percentages. The data were analyzed using a chi-square test or Student’s t test. The association between operative mortality and the different risk factors was examined using logistic regression, and results are expressed as odds ratio (OR) with a 95% confidence interval (CI). Among risk factors, those with a *P*-value ≤0.05 were selected for the multivariate analyses. The multivariate models were constructed from the Hosmer-Lemeshow approach. A *P* value < 0.05 was considered statistically significant. All statistical analyses were performed using SPSS version 21.0 for Windows (SPSS Inc., Chicago, IL, USA).

## Results

### Patient characteristics

The demographic and operative data of the 2 groups are shown in Table 1, and the distribution of the NCEP-ATP III criteria used to define MetS is presented in Table 2. About 49.4% MetS patients do not have DM as part of their MetS criteria. There were no significant differences between the 2 groups for age, gender, various concomitant diseases, severity of coronary artery disease, operative data, echocardiographic findings or the new logistic EuroSCORE II (a measure of the operative risk). However, as expected, the rates of DM, hypertension and obesity were significantly higher in patients with MetS than in those without MetS (*P* < 0.05 for each comparison). Patients with MetS exhibited significantly higher fasting glycemia, hyperuricemia, and plasma triglyceride levels than those without MetS. Moreover, there was a significant difference in the number of patients with NYHA heart failure class III/IV between the 2 groups. The mean number of anastomoses was 2.50 ± 0.93 in the non-MetS group and 2.75 ± 0.89 in the MetS group (*P* = 0.059).

**Table 1:** Comparison of characteristics based on the presence or absence of metabolic syndrome

		Metabolic Syndrome		
Variables	All Patients (*n* = 190)	Absent (*n* = 103)	Present (*n *= 87)	*P* value
Age, yrs	62.7 ± 9.2	62.6 ± 9.8	62.7 ± 8.4	0.964
Female gender	20 (10.5%)	7 (6.8%)	13 (14.9%)	0.068
BMI,kg/m^2^	24.9 ± 4.2	23.0 ± 3.2	27.1 ± 4.1	*P*<0.0001
Expected operative risk (by EuroSCORE II) (%)	3.5 ± 6.7	3.2 ± 6.9	4.0 ± 6.9	0.399
NYHA heart failure class (III/IV), n(%)	66 (34.7%)	27 (26.2%)	39 (44.8%)	0.007
Risk factors and concomitant diseases				
Smoking				
Never	81 (42.6%)	43 (41.7%)	38 (43.7%)	0.789
Former	35 (18.4%)	19 (18.4%)	16 (18.4%)	0.992
Current	74 (38.9%)	41 (38.9%)	33 (37.9%)	0.792
Obesity (BMI>25 kg/m^2^)	18 (9.5%)	4 (3.9%)	14 (16.1%)	0.004
Hypertension	108 (56.8%)	36 (35.0%)	72 (82.8%)	P<0.0001
Diabetes mellitus	63 (33.2%)	19 (18.4%)	44 (50.6%)	*P*<0.0001
Lipid profile				
HDL cholesterol, mmol/L	1.05 ± 0.69	1.14 ± 0.9	0.94 ± 0.24	0.038
LDL cholesterol, mmol/L	2.97 ± 1.16	2.68 ± 1.06	3.30 ± 1.20	*P*<0.001
Triglycerides, mmol/L	1.6 ± 0.9	1.27 ± 0.43	2.0 ± 1.15	*P*<0.0001
Fasting glycaemia, mmol/L	6.5 ± 2.8	5.6 ± 1.9	7.6 ± 3.2	*P*<0.0001
CK-MB, μg/L	21.3 ± 33.9	18.6 ± 15.9	24.5 ± 47.1	0.231
Hyperuricemia	50 (26.3%)	22 (21.4%)	28 (32.2%)	0.091
COPD	6 (3.2%)	2 (1.9%)	4 (4.6%)	0.297
Peripheral vascular disease	17 (8.9%)	6 (5.8%)	11 (12.6%)	0.101
History of stroke	15 (7.9%)	6 (5.8%)	9 (10.3%)	0.250
Renal dysfunction (creatinine>150 μmol/L)	12 (6.3%)	4 (3.9%)	8 (9.2%)	0.134
Used of mechanical ventilation	11 (5.8%)	5 (4.9%)	6 (6.9%)	0.548
Details of coronary artery disease				
Left main stenosis ≥50%	57 (30%)	31 (30.1%)	26 (29.9%)	0.975
2-vessel disease(≥75% stenosis)	54 (28.4%)	26 (25.2%)	28 (32.2%)	0.291
3-vessel disease(≥75% stenosis)	119 (62.6%)	63 (61.2%)	56 (64.4%)	0.649
Recent myocardial infarction (<30 days)	69 (36.3%)	38 (36.9%)	31 (35.6%)	0.857
Atrial fibrillation	10 (5.3%)	7(6.8%)	3 (3.4%)	0.303
Previous PCI	24 (12.6%)	17 (16.5%)	7 (8.0%)	0.080
Echocardiographic findings				
LV ejection fraction (%)	39.8 ± 6.4	39.3 ± 6.3	40.4 ± 6.6	0.237
LV diastolic dimension (mm)	56.8 ± 6.9	56.5 ± 7.4	57.1 ± 6.2	0.505
Pulmonary hypertension	59 (31.1%)	35 (34.0%)	24 (27.6%)	0.343
Mitral valve insufficiency	96 (50.5%)	52 (50.5%)	44 (50.6%)	0.990
Left ventricular aneurysm	31 (16.3%)	20 (19.4%)	11 (12.6%)	0.208
Operative data				
Urgent/emergency surgery	7 (3.7%)	3 (2.9%)	4 (4.6%)	0.705
On-pump bypass surgery	54 (28.4%)	31 (30.1%)	23 (26.4%)	0.577
IMA used as grafts	161 (84.7%)	85 (82.5%)	76 (87.4%)	0.356
Saphenous vein used as grafts	180 (94.7%)	96 (93.2%)	84 (96.6%)	0.303
Aneurysmectomy	10 (5.3%)	8 (7.9%)	2 (2.3%)	0.183
Mitral valve surgery	17 (8.9%)	10 (9.7%)	7 (8.0%)	0.689
No. of distal anastomosis	2.6 ± 0.9	2.5 ± 0.93	2.75 ± 0.89	0.059

Mean ± SD for continuous variables are shown

(*BMI* = body mass index, *NYHA* = New York Heart Association, *EuroSCORE* = European system for cardiac operatic risk evaluation, *COPD* = chronic obstructive pulmonary disease, *PCI* = percutaneous coronary intervention, *HDL* = high-density lipoprotein, *LDL* = low-density lipoprotein, *CK-MB* = creatine kinase-myocardial band, *LV* = left ventricular, *IMA* = internal mammary artery, *SD* = standard deviation)

**Table 2:** Prevalence and distribution of the NCEP-ATPIII criteria for metabolic syndrome of 190 patients

		Metabolic Syndrome		
	All Patients (*n* = 190)	Absent (*n* = 103)	Present (*n* = 87)	*P* value
Obesity (BMI>25 kg/m^2^)	94 (49.5%)	22 (21.4%)	72 (82.8%)	*P*<0.0001
Fasting glycemia ≥6.1 mmol/L or (and) Diabetes mellitus	102 (53.6%)	30 (29.1%)	72 (82.8%)	*P*<0.0001
Triglycerides ≥1.69 mmol/L	57 (30%)	7 (6.8%)	50 (57.5%)	*P*<0.0001
HDL cholesterol (<1.04 mmol/L in men and<1.29 mmol/L in women)	107 (56.3%)	39 (37.9%)	68 (78.2%)	*P*<0.0001
Hypertension	108 (56.8%)	36 (35.0%)	72 (82.8%)	*P*<0.0001
0 criteria	15 (7.9%)	15 (14.6%)	0	–
1 criteria	41 (21.6%)	41 (39.8%)	0	–
2 criteria	47 (24.7%)	47 (45.6%)	0	–
3 criteria	34 (17.9%)	0	34 (39.1%)	–
4 criteria	33 (17.4%)	0	33 (37.9%)	–
5 criteria	20(10.5%)	0	20 (23.0%)	–

(*BMI* = body mass index, *HDL* = high-density lipoprotein, *NCEP-ATP* III=National Cholesterol Education Program Adult Treatment Panel III)

### Morbidity and mortality

Postoperative morbidity and mortality are presented in Table 3. The overall in-hospital mortality rate was 7.9 (15 of 190 patients). There were statistically significant differences between the 2 groups in the rates of in-hospital mortality, acute renal failure, ventricular fibrillation, septicemia and new intra-aortic balloon pump (IABP) insertion (all *p* values < 0.05). There was a trend toward higher rates of prolonged ventilation in MetS patients (9.2% vs. 3.9%, *p* = 0.05), but it did not reach statistical significance.

**Table 3:** Comparison of in-hospital outcomes based on the presence or absence of metabolic syndrome

		Metabolic Syndrome		
Variables	All Patients (*n* = 190)	Absent (*n* = 103)	Present (*n* = 87)	*P* value
In-hospital mortality	15 (7.9%)	4 (3.9%)	11 (12.6%)	0.026
Duration of ICU Stay>48 h	67 (35.3%)	32 (31.1%)	35 (40.2%)	0.188
Ventilation Time>48 h	25 (13.2%)	9 (8.7%)	16 (18.4%)	0.05
Reintubation	12 (6.3%)	4 (3.9%)	8 (9.2%)	0.134
Intra- and postoperative use of IABP	16 (8.4%)	4 (3.9%)	12 (13.8%)	0.014
Septicemia	7 (3.7%)	1 (1.0%)	6 (6.9%)	0.049
Ventricular fibrillation	14 (7.4%)	4 (3.9%)	10 (11.5%)	0.045
Acute renal failure	21 (11.1%)	6 (5.8%)	15 (17.2%)	0.012
Stroke	4 (2.1%)	1 (1.0%)	3 (3.4%)	0.334
LCOS	12 (6.3%)	4 (3.9%)	8 (9.2%)	0.134
Myocardial infarction	8 (4.2%)	2 (1.9%)	6 (6.9%)	0.145
New atrial fibrillation	15 (7.9%)	8 (7.8%)	7 (8.0%)	0.943
Gastrointestinal hemorrhage	7 (3.7%)	2 (1.9%)	5 (5.7%)	0.25

(*ICU* = intensive care unit, *IABP* = intra-aortic balloon pump, *LCOS* = low cardiac output syndrome)

**Fig. 1:**
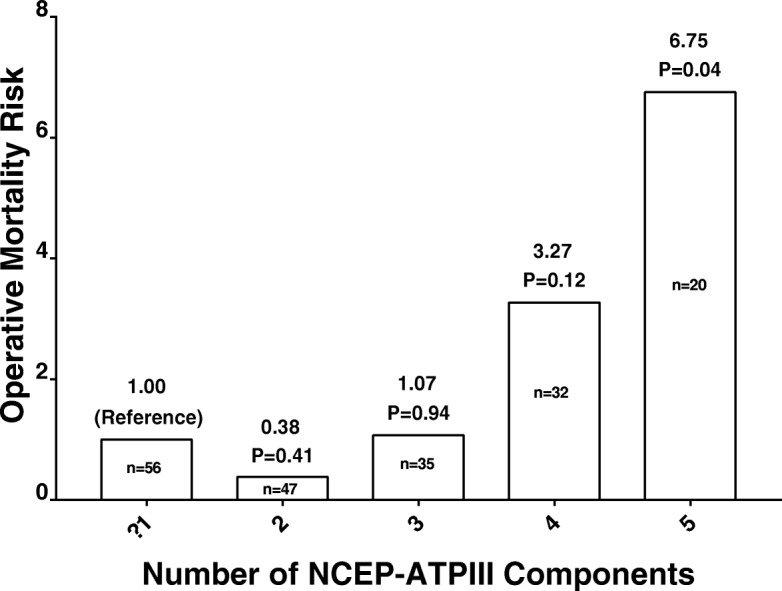
The Effect of the Number of Metabolic Syndrome Components on Operative Mortality Risk

In univariate analysis, MetS was associated with a 3.58-fold increase in the risk of mortality. There was no indication of a cumulative effect in relation to the number of NCEP-ATP III components of MetS (Fig. 1). Among the other risk factors, an age > 70, history of stroke, preoperative renal dysfunction, patients with NYHA class III/IV, urgent or emergent operative status, on-pump bypass surgery and the new logistic EuroSCORE II were significantly associated with higher in-hospital mortality rates (Table 4).

**Table 4:** Univariate analysis of potential risk factors for operative mortality

Risk factors	Odds ratio	95% CI	*P* value
Female gender	1.71	0.21–13.70	0.616
Age>70	5.11	1.73–15.11	0.003
Expected operative risk (by EuroSCORE II) (%)	1.19	1.09–1.30	<0.0001
Metabolic syndrome	3.58	1.10–11.69	0.034
Obesity (BMI>25 kg/m^2^)	2.17	0.71–6.60	0.174
Hypertension	1.15	0.39–3.38	0.797
Diabetes mellitus	2.49	0.86–7.22	0.092
HDL cholesterol (<1.04 mmol/L in men and<1.29 mmol/L in women)	5.45	1.77–16.78	0.03
Triglycerides ≥1.69 mmol/L	1.14	0.40–3.28	0.808
Fasting glycemia ≥6.1 mmol/L	4.33	1.32–14.14	0.015
Previous percutaneous coronary intervention	1.83	0.48–7.04	0.377
Smoking	2.16	0.66–7.05	0.202
NYHA heart failure class (III/IV)	6.00	1.83–19.68	0.003
Peripheral vascular disease	2.88	0.73–11.41	0.133
Chronic obstructive pulmonary disease	2.43	0.27–22.25	0.432
History of stroke	5.42	1.48–19.83	0.011
Renal dysfunction (creatinine>150 μmol/L)	7.59	1.98–29.18	0.003
Left main stenosis ≥50%	2.19	0.75–6.35	0.15
3-vessel disease (≥75% stenosis)	1.71	0.52–5.58	0.377
LV ejection fraction (%)	0.94	0.87–1.02	0.166
LV diastolic dimension (mm)	1.01	0.94–1.09	0.804
Pulmonary hypertension	2.78	0.96–8.07	0.06
Mitral valve surgery	2.88	0.73–11.41	0.133
Urgent/emergency surgery	43.25	7.45–251.23	<0.0001
On-pump bypass surgery	4.33	1.46–12.85	0.008
IMA used as grafts	0.7	0.18–2.64	0.597
Mitral valve insufficiency	1.52	0.52–4.44	0.447
No. of distal anastomosis	1.17	0.66–2.08	0.59

(*95% CI* = 95% confidence interval, *BMI* = body mass index, *EuroSCORE* = European system for cardiac operatic risk evaluation, *HDL* = high-density lipoprotein, *LV* = left ventricular, *IMA* = internal mammary artery)

In multivariate logistic regression analysis, the presence of MetS was strongly associated with an increased incidence of in-hospital mortality (OR: 5.99, 95% CI: 1.02–35.15, *P* = 0.047). Ages > 70 were also independent predictors of in-hospital mortality (Table 5). However, DM, hypertension, obesity, and the 5 components of MetS included in the NCEP-ATP III definition did not increase mortality after CABG during hospitalization (all *P* values < 0.05). In patients without DM, MetS was associated with higher risk of mortality whether DM was part of MetS or not (11.6% vs. 2.4%; *p* = 0.044), while in diabetes patients MetS was not associated with higher risk (13.6% vs. 10.5%; *p* = 0.73). Table 6 presents the causes of death. Deaths of cardiac origin accounted for 40% of the deaths. There were no significant differences with respect to the causes of death among patients with or without MetS.

**Table 5:** Multivariable analysis of potential risk factors for operative mortality

Risk factors	Odds ratio	95% CI	*P* value
Metabolic syndrome	5.99	1.02–35.15	0.047
Age>70	11.51	2.22–59.70	0.004
History of stroke	4.41	0.69–28.25	0.110
Renal dysfunction (creatinine>150 μmol/L)	2.11	0.16–27.43	0.569
NYHA heart failure class (III/IV)	2.32	0.40–13.48	0.350
Urgent/emergency surgery	2.24	0.11–44.12	0.596
On-pump bypass surgery	4.55	0.89–23.13	0.068
New EuroSCORE II	1.12	0.10–1.26	0.06

(*95% CI* = 95% confidence interval, *NYHA* = New York Heart Association; *EuroSCORE* = European system for cardiac operatic risk evaluation)

**Table 6:** Postoperative cause of mortality for patients undergoing CABG according to metabolic syndrome status

		Metabolic syndrome		
Cause	All Patients	Absent	Present	p Value
Cardiac	6	1	5	0.095
Neurologic	1	0	1	0.458
Septicemia	2	1	1	1.000
Respiratory	1	0	1	0.458
Multiple system failure	4	1	3	0.334
Renal	1	1	0	1.000

(*CABG* = coronary artery bypass grafting)

## Discussion

MetS increases the risk of developing coronary artery disease (CAD) and is also more prevalent among patients who undergo CABG. MetS has been reported to occur in up to 13% of the general population in China [17]. However, in our study, the prevalence of MetS of 45.8% was consistent with that of previous studies in which MetS and CABG were investigated [5, 12, 18].

Several studies have reported an association between postoperative outcomes and MetS [6, 7, 12, 13, 18]. According to these studies, patients with MetS have increased risks morbidity and mortality after CABG, both overall and from CVD. One of the negative effects of MetS is a change in the structure and function of the left ventricle [19]. As Azevedo et al. found, increased severity of MetS is associated with increasingly compromised structure and function of the heart [20]. Yazicio et al. also reported that more severely impaired LV systolic function after acute MI may contribute to the higher morbidity and mortality observed in patients with MetS [10]. However, whether MetS still has an adverse effect on postoperative prognosis among patients with LV dysfunction who undergo CABG has not been demonstrated. This is the first study to delineate the role of MetS in operative mortality and complications in these patients. In our study, we found that, in line with previous reports, MetS is an important predictor for higher operative morbidity and mortality in CABG patients with impaired LV systolic function.

The components of MetS, such as diabetes, hypertension and obesity, have been reported to be associated with a higher incidence of operative mortality after CABG. When analyzing 41,663 patients with diabetes, Carson JL et al. found increased morbidity and morbidity after CABG in diabetic patients during hospitalization when compared with nondiabetic patients [4]. Despite the lack of convincing evidence in the literature, obesity is often considered to be a significant risk factor for postoperative mortality when selecting candidates for CABG [21–23]. Moulton et al. concluded that those with a history of hypertension have an increased frequency of immediate post-operative complications and an increased 2-year mortality after CABG [24]. Aronson S et al. also reported that isolated systolic hypertension is associated with increased perioperative cardiovascular morbidity in coronary artery surgery patients [25]. However, patients with DM, obesity and hypertension did not have increased morbidity and mortality independently in our study. The discordant results may result from disagreement among the criteria adopted or the characteristics of the populations and the methodological strategies used.

We also found the presence of both MetS and DM significantly increased the risk of operative mortality among patients with impaired LV systolic function, whereas patients without MetS were not at higher risk. These results demonstrated that the effect of MetS on operative mortality was not a single effect of DM, but the aggregation of multiple factors, such as hypertension, hyperglycemia, and obesity, which acted as a combined risk factor for operative mortality. The clustering of cardiovascular risk factors in MetS demonstrated that the multiple complex metabolic reactions involved in glycotoxicity, lipotoxicity, altered insulin signaling, increased cytokine activity and interstitial deposition of triacylglycerol may directly or indirectly impact myocardial function, and then reduce survival in MetS patients with LVEF<50%. Moreover, we found that the distributions of the causes of death were similar among patients with or without MetS, and about 40% deaths were of cardiac origin. Regarding postoperative complications, there were differences in the percentages of patients with ventricular fibrillation, septicemia and renal failure after CABG between the two groups.

This study provides further evidence that MetS is a prevalent and important risk factor for operative mortality after CABG in patients with impaired with LV systolic function. Furthermore, the components of MetS have synergistic effect in postoperative mortality among patients with EF<50%. These findings have major clinical implications. For instance, multifactorial intervention in patients with MetS who are referred to CABG is required for improving poor prognosis, including optimal control of lipids, blood pressure, blood glucose and body weight. It should be noted, however, that many patients with MetS are not being treated appropriately [12]. There is a definite need for clinics to focus on developing tools to reduce conditions associated with MetS. These tools include early identification, education, lifestyle modifications, and pharmacological interventions. In addition, the identification of MetS might be helpful for classifying high-risk patients, improving risk stratification for CABG patients and assessing the prognosis of CVD. Further large-scale and long-term studies are needed to determine whether MetS is responsible for the increased mortality after CABG surgery, especially in patients with impaired LV systolic function. Further studies are needed to determine whether perioperative medications might be effective in reducing mortality in MetS patients after CABG surgery. In addition, Further studies are necessary to clarify the mechanisms that the components of MetS work synergistically to increase the risk of operative mortality.

### Limitations

This was a retrospective single-center study on ethnic Chinese patients. Its retrospective nature and the small number of patients limit the validity of the clinical outcome. However, the use of specific statistical evaluations enabled relatively precise risk and outcome assessments and comparisons. Although larger samples would be needed to produce more accurate and convincing results, we believe that our study already presents interesting findings related to postoperative outcomes. In addition, because waist circumference data were not available, we selected a BMI > 25 kg/m^2^ as the cutoff point for obesity based on the results of a previous study on the relationship between BMI, waist circumference, and obesity in a Chinese population. If waist circumstances had been used to define obesity in the detection of MetS, more specific results might have been obtained.

## Conclusions

In conclusion, patients with MetS have a higher risk for operative mortality in patients with impaired LV systolic function who undergo CABG. The components of MetS, such as diabetes, hypertension and obesity did not increase operative mortality independently in our study. The effect of MetS on operative mortality was the aggregation of multiple factors, which work synergistically to increase the risk of operative mortality. In addition, the identification of MetS might be helpful for classifying high-risk patients, improving risk stratification for CABG patients with LVEF<50% and assessing the prognosis of CVD.

## Abbreviations

BMI: Body mass index
CABG: Coronary artery bypass grafting
CAD: Coronary artery disease
CK-MB: Creatinine kinase-MB
COPD: Chronic obstructive pulmonary disease
CVD: Cardiovascular disease
DM: Diabetes mellitus
EF: Ejection fractions
EuroSCORE: European system for cardiac operatic risk evaluation
HDL: High-density lipoprotein
IABP: Intra-aortic balloon pump
ICU: Intensive care unit
IMA: Internal mammary artery
LCOS: Low cardiac output syndrome
LDL: Low-density lipoprotein
LV: Left ventricular
MetS: Metabolic syndrome
MI: Myocardial infarction
NCEP-ATP III: National Cholesterol Education Program Expert Panel on Detection, Evaluation, and Treatment of High Blood Cholesterol in Adults
NYHA: New York Heart Association
PCI: Percutaneous coronary intervention
RRs: Relative risks
SD: Standard deviation
